# A retrospective cohort study on the relationship between frailty and healthcare outcomes

**DOI:** 10.1016/j.tjfa.2025.100053

**Published:** 2025-05-23

**Authors:** Jinmyoung Cho, Joanne Salas, Jeffery F. Scherrer, George Grossberg

**Affiliations:** a Department of Family and Community Medicine, Saint Louis University School of Medicine, St. Louis, MO, USA; b AHEAD Institute, Saint Louis University, St. Louis, MO, USA; c Department of Psychiatry and Behavioral Neuroscience, Saint Louis University School of Medicine, St. Louis, MO, USA

**Keywords:** Frailty, Older patients, Healthcare services

## Abstract

**Background:**

Frailty increases vulnerability for adverse outcomes in older adults. Characterizing the prevalence and distribution of frailty can help guide healthcare service decision-making and policy.

**Objectives:**

This study evaluated the association between frailty and healthcare utilization and interactions by demographic characteristics.

**Design:**

Using electronic health records (2018–2022), we conducted a retrospective cohort study with 355,266 patients ≥65 years of age who had ≥2 ambulatory office visits in separate years in the 4-year baseline period (2018–2021). The Gilbert Frailty Index (GFI) was calculated (low vs. intermediate vs. high) using ICD-10 codes. One-year utilization outcomes in 2022 included high outpatient clinic utilizations (OCU), inpatient (IP), emergency department (ED), and nursing home (NH) admissions. Fully adjusted log-binomial regression models were calculated overall and by race (White vs. Black), age groups, and gender.

**Results:**

The sample was 74.5(±7.5) years of age, 57.7 % female, 89.2 % White, and 13.5 % categorized as GFI high. After adjustment for covariates, GFI high had the highest risk for all outcomes (RR=3.31 for IP; 2.77 for ED; 4.26 for NH; 1.60 for high OCU). We observed significant interactions by race, gender, and age for some outcomes. Effects of GFI high vs. low were larger for White (IP, ED, & high OCU), female patients (ED & high OCU), and younger patients (IP). Conversely, the effects of GFI high vs. low were strongest in older patients for ED, IP and high OCU.

**Conclusions:**

Monitoring frailty and paying attention to patient’s demographic characteristics is needed to best estimate associations between frailty and healthcare utilization.

## 1 Introduction

Frailty is defined as “the state of increased vulnerability”[[1], [2], [3]] resulting from a decline in physiological capacity in multiple organ systems as individuals age [[4], [5], [6]]. The growing aging population has led to a rise in the prevalence of frailty among older adults [7,8]. Frail older adults often require significant attention from healthcare systems. Frailty is associated with increased mortality, greater multimorbidity, increased medication use, and a higher risk for hospitalization [[9], [10], [11], [12], [13]], all of which would contribute to elevated healthcare costs [14].

Due to its significant concern for the healthcare of older adults, prior research has developed various tools and instruments to assess frailty. Two models have been well-established: frailty phenotype and frailty index. The former focuses on physical signs and symptoms, while the latter quantifies frailty based on the accumulation of deficits relevant to age-related changes in physical, cognitive, mental, and functional health and diseases [4,[15], [16], [17]]. Although more than 65 frailty assessments have been developed based on these two approaches, most rely on in-person evaluations or patient-reported functional measurements [18,19]. This dependency poses challenges in direct clinical care settings and limits their applicability at the population level.

While frailty becomes an increasingly critical factor for medication use, health outcomes, and healthcare utilization among older adults [17], a systematic approach to assessing frailty is needed for implementing targeted interventions and care planning, particularly for patients likely to become high healthcare utilizers [20]. Using administrative data from health insurance claims, a number of studies used the Claims-based Frailty Index (CFI) to examine the treatment effects of interventions among frail patients in clinical trials [21]. Although the CFI is considered a simple tool for assessing frailty, it still requires manual assessment, which is time consuming and creates potential errors [22].

Recently, Gilbert et al. developed the Frailty Index (GFI) score with ICD-9/10 codes to identify older patients at risk of adverse healthcare outcomes. The GFI includes 109 diagnostic items, and each has different weights based on associations with comorbidities, functional limitations, cognitive decline, and geriatric syndromes, resulting in a composite index ranging from 0 to 173.2 [23]. The GFI is advantageous because it can be easily incorporated into hospital information systems wherever the coding system is available, removing the burdens of manual scoring methods [22]. Due to these benefits, the GFI has been validated across various patient populations [[24], [25], [26], [27], [28], [29], [30]]. While the GFI was initially developed for use in hospitalized older patients, it can be applied to broader clinical settings with ICD-9/10 codes. Several recent studies have demonstrated its potential as a pragmatic tool for identifying frailty risk outside acute settings [26,27]. Given the growing emphasis on preventive care, applying GFI with ICD-9/10 codes to general older patients may be beneficial to detect early-stage of frailty before hospitalization occurs. Therefore, this study extends prior validation efforts to apply the GFI to older patients who are regularly engaged with ambulatory care. Furthermore, the impact of demographic characteristics (e.g., gender, race-ethnicity, advanced age group) on the GFI has not been explored yet, despite evidence showing significant racial-ethnic variations in frailty using phenotype or accumulation deficits models [31,32]. For instance, a study using a nationally representative profile found that the prevalence of frailty was estimated with 22.9 % for Black individuals and 24.6 % for Hispanic individuals, compared to less than 14 % for White or other racial-ethnic groups [33]. Additionally, direct associations between the GFI and hospital service utilization among patients from acute care settings have not been sufficiently studied. Therefore, the purpose of this work is to evaluate the association between the GFI and healthcare utilization as well as to assess how this association is moderated by demographic characteristics (i.e., age groups, gender, and race).

## 2 Methods

De-identified medical record data from a local healthcare system’s Virtual Data Warehouse (VDW) from calendar years 2018 to 2022 were used in this retrospective cohort analysis. The local healthcare system is a member site of the Health Care Systems Research Network (HCSRN) (www.hcsrn.org). Data used to create variables in the current study included ICD-10-CM diagnostic codes, Current Procedural Terminology Codes (CPT), pharmacy orders/prescriptions, clinic or encounter type, and demographics. The VDW additionally includes vital signs, laboratory results, provider type, social history-related information (e.g. tobacco use), and referral data. From 2018 to 2022, the VDW includes over 3.25 million patients from birth to > 90 years of age who have utilized any type of healthcare service in the healthcare system. Utilization includes a wide range of encounter types (e.g., outpatient or ambulatory visits, inpatient stays, same-day surgeries, primary care or specialist office visits, virtual encounters, lab or procedure only visits, etc.) with all previously mentioned data recorded at each visit. The VDW includes medical record data from academic and non-academic ambulatory and inpatient clinical encounters in the local healthcare system, which covers rural and urban locations from the Midwest, specifically St Louis, MO metropolitan area, mid-Missouri, southern Illinois, Oklahoma City metropolitan area, and southern Wisconsin. The local Institutional Review Board reviewed our research using the VDW as exempt. Additional details regarding the VDW have been published [[34], [35], [36]].

### 2.1 Eligibility

This retrospective cohort analysis defined a baseline period, 2018 to 2021, where the main exposure and all covariates were measured. The index date was 1/1/2022, and all patients had a one-year follow-up period in 2022 to measure outcomes. Patients must have been at least 65 years old at index and had at least two ambulatory office visits, occurring in separate calendar years, in the baseline period to identify regular users of the health care system. Ambulatory clinic visits used in eligibility could be any ambulatory visit to primary care physicians or specialists, but those related to emergency department encounters, or same-day surgeries were not counted towards eligibility. Patients were excluded if they were admitted or living in a nursing home at the start of 2022. The final analytic sample included 355,266 patients after removing patients with missing gender (*n* = 5) or missing race (*n* = 6562) (See Supplement Figure 1).

### 2.2 Exposure

The primary exposure variable was the Gilbert Frailty Index (GFI), measured in the 4-year baseline period. The GFI is a validated hospital frailty risk score derived from the presence of ICD-10 diagnostic codes documented during any type of healthcare encounter in electronic health records. It includes 109 diagnostic items, each assigned a weight based on its statistical association with frailty-related outcomes, such as dementia, falls, urinary incontinence, and functional impairments. These conditions are assigned weights established by Gilbert et al. and summed to generate a total score, where a higher total score indicates higher frailty risk [23]. Per published cutoffs, the GFI score was categorized into low risk (<5), intermediate risk (5–15), and high risk (>15). Supplement Table 1 contains a general description of the GFI and Supplement Table 2 lists each condition and its assigned point value.

**Table 1:** Sample characteristics and outcomes of patients ≥ 65 years old (*n* = 355,266).

Characteristics	N ( %) or Mean(±sd)
Demographics	
Age, mean(±sd)	74.5 (±7.5)
Age category	
65–74	204,737 (57.6 %)
75–84	108,831 (30.6 %)
≥85	41,698 (11.7 %)
Female gender	204,982 (57.7 %)
Race	
White	316,757 (89.2 %)
Black	31,547 (8.9 %)
Other	6962 (1.9 %)
*Gilbert Frailty Index (GFI) (2018–2021)*	
GFI, mean(±sd)	7.1 (±8.1)
GFI group	
Low	186,582 (52.5 %)
Intermediate	120,339 (33.9 %)
High	48,345 (13.6 %)
*Outcomes in 2022*	
Any inpatient (IP) admission	30,554 (8.6 %)
Any emergency (ED) admission	48,466 (13.6 %)
Nursing home (NH) admission	11,819 (3.3 %)
High Outpatient clinic utilization (OCU)	141,233 (39.7 %)
*Other covariates (2018–2021)*	
Smoking	75,779 (21.3 %)
Number of medication classes RX, mean(±sd)	5.7 (±4.0)

**Table 2:** Covariates and outcomes by GFI group (*n* = 355,266).

	Low (*n* = 186,582)	Intermediate (*n* = 120,339)	High (*n* = 48,345)	p-value
Covariates				
Age category				<0.0001
65–74	117,914 (63.2)	67,519 (56.1)	19,304 (39.9)	
75–84	52,613 (28.2)	38,485 (32.0)	17,733 (36.7)	
≥85	16,055 (8.6)	14,335 (11.9)	11,308 (23.4)	
Female gender	103,905 (55.7)	70,883 (58.9)	30,194 (62.5)	<0.0001
Race				<0.0001
White	168,971 (90.6)	106,442 (88.4)	41,344 (85.5)	
Black	13,859 (7.4)	11,515 (9.6)	6173 (12.8)	
Other	3752 (2.0)	2382 (2.0)	828 (1.7)	
Smoking	30,234 (16.2)	30,436 (25.3)	15,109 (31.3)	<0.0001
Number of medication classes, mean(±sd)	3.6 (±3.3)	7.3 (±3.4)	9.7 (±2.8)	<0.0001
Outcomes in 2022				
Any IP admission	8339 (4.5)	12,399 (10.3)	9816 (20.3)	<0.0001
Any ED admission	14,528 (7.8)	20,767 (17.3)	13,171 (27.2)	<0.0001
NH admission	2402 (1.3)	5233 (4.4)	4184 (8.6)	<0.0001
High OCU	50,208 (26.9)	63,771 (53.0)	27,254 (56.4)	<0.0001

### 2.3 Outcomes

Healthcare utilization outcomes, measured in 2022, included any inpatient admission (IP, yes or no), an emergency department admission (ED, yes or no), indication of nursing home admission or stay (NH, yes or no), and high ambulatory office outpatient utilization (OCU, yes or no). IP admissions were indicated by encounter types for inpatient stays; similarly, ED admissions were indicated by encounter types for emergency department visits. High OCU was defined as at least four unique ambulatory, outpatient clinic encounters in the outcome year. This cutoff was chosen as it could signify, on average, at least one visit quarterly. Finally, NH utilization or admission was defined by either: a) an encounter type of institutional stay in a nursing home; b) CPT codes 99304–99310, 99315 or 99316; or ICD-10-CM diagnosis code of Z02.2 or Y92.12.

### 2.4 Covariates

Based on clinical relevance and previous research demonstrating their association with both frailty and healthcare utilization [21,23,37], covariates included demographics, smoking history, and number of prescription drug classes prescribed. Demographic variables were age at index (65–74, 75–84, ≥85), race (white, black, other), and gender (male, female). Prescription drug class and smoking were measured in the 4-year baseline period. Smoking or nicotine dependence was measured by “current smoker” status in the social history or ICD-10-CM diagnostic code for nicotine dependence (See Supplement Table 1). Prescription drug class was the count of the number of major drug classification groups ever prescribed in the baseline period using Medi-Span generic product identifier (e.g., Groups 01–16: Anti-infective agents; 17–20: Biologicals; 21: Antineoplastic agents; 22–30: Endocrine and Metabolic drugs, etc.; see Supplement Table 1 for major classes of drug classification).

### 2.5 Analytic approach

All analyses were performed using SAS v9.4 (SAS Institute, Cary, NC). Means (±standard deviation) and frequencies and percents summarized all study variables. The distribution of covariates and outcomes were compared between the three GFI groups using chi-square tests for categorical variables and a crude, negative binomial model for number of prescription classes. Crude and adjusted log-binomial models estimated the relative risk (RR) and 95 % confidence interval (CI) for the relationship of GFI group and each healthcare utilization outcome. Overall, adjusted models controlled for age, race, gender, smoking, and number of medication classes prescribed. Adjusted models were then stratified by White vs. Black race, gender (male vs. female) and age category. An interaction term of each of these characteristics and GFI in overall adjusted models assessed whether RR’s were different between strata.

## 3 Results

### 3.1 Characteristics of the analytic samples

Overall cohort characteristics are shown in Table 1. Patients were, on average, 74.5(±7.5) years of age, 57.7 % were female, 89.2 % were White and 8.9 % were Black race. Mean GFI score was 7.1(±8.1) with about half of patients with GFI low (52.5 %), one-third (33.9 %) intermediate, and 13.6 % with high. About one in five patients were smokers (21.3 %). In 2022, 8.6 % of the sample had an IP admission, 13.6 % had an ED admission, 3.3 % had a NH admission and 39.7 % had high OCU. Table 2 shows the relationship of GFI group with covariates and outcomes. A greater proportion of GFI high patients compared to lower risk groups were at least 85 years old, female, black and smokers. Similarly, each healthcare outcome was more prevalent as GFI risk increased.

### 3.2 Associations between healthcare outcomes and GFI

Table 3 shows crude and adjusted models for the relationship of each outcome with GFI. In adjusted models, compared to patients with GFI low, patients with GFI intermediate had approximately double the risk of IP (RR=1.95; 95 %CI=1.89–2.01) or ED admissions (RR=1.93; 95 %CI=1.89–1.98). This risk increased when comparing high to low patients for IP (RR=3.31; 95 %CI=3.19–3.43) and ED admissions (RR=2.77; 95 %CI=2.69–2.84). For NH admissions, GFI intermediate and high compared to GFI low had a 2.65 and 4.26, respectively, increased risk of admission. Finally, GFI intermediate and high compared to GFI low showed similar risks of high OCU (RR=1.60 and 1.63, respectively).

**Table 3:** IP, ED, and NH admission and high OCU outcomes before and after controlling for confounding. Results from log-binomial models, relative risk (95 % CI) (*n* = 355,266)^a^.

Outcome	GFI Low RR (95 % CI)	GFI Intermediate RR (95 % CI)	GFI High RR (95 % CI)
IP admission			
Crude	1.00	2.31 (2.24–2.37)	4.54 (4.42–4.67)
Adjusted	1.00	1.95 (1.89–2.01)	3.31 (3.19–3.43)
*ED admission*			
Crude	1.00	2.22 (2.17–2.26)	3.50 (3.42–3.57)
Adjusted	1.00	1.93 (1.89–1.98)	2.77 (2.69–2.84)
*NH admission*			
Crude	1.00	3.38 (3.22–3.54)	6.72 (6.40–7.06)
Adjusted	1.00	2.65 (2.51–2.80)	4.26 (3.99–4.56)
*High OCU*			
Crude	1.00	1.97 (1.95–1.99)	2.10 (2.07–2.12)
Adjusted	1.00	1.63 (1.631–1.65)	1.60 (1.58–1.62)

Note: RR = relative risk; CI = confidence interval; GFI = Gilbert Frailty Index; IP = Inpatient; ED = Emergency Department; NH = Nursing Home; OCU = Outpatient Clinic Utilization.

a Fully adjusted models include age, gender, race, smoking, and number of medication classes.

### 3.3 Demographic characteristics, GFI risks, and healthcare utilization outcomes

Fig. 1a shows age, gender, and White vs. Black race stratified results for IP admissions. Results showed that race and age modified the association of GFI and IP admission but gender did not. For both GFI intermediate and GFI high vs. GFI low, effects were larger for White vs. Black patients (RR=2.01 vs. 1.55 and RR=3.45 vs. 2.42, respectively). GFI intermediate vs. GFI low had similar effects for all age groups; however, the effect of GFI high vs. GFI low in age groups < 85 were stronger larger than ≥85 years old (RR=3.71 and 3.32 vs. 2.44).

**Figure 1a:**
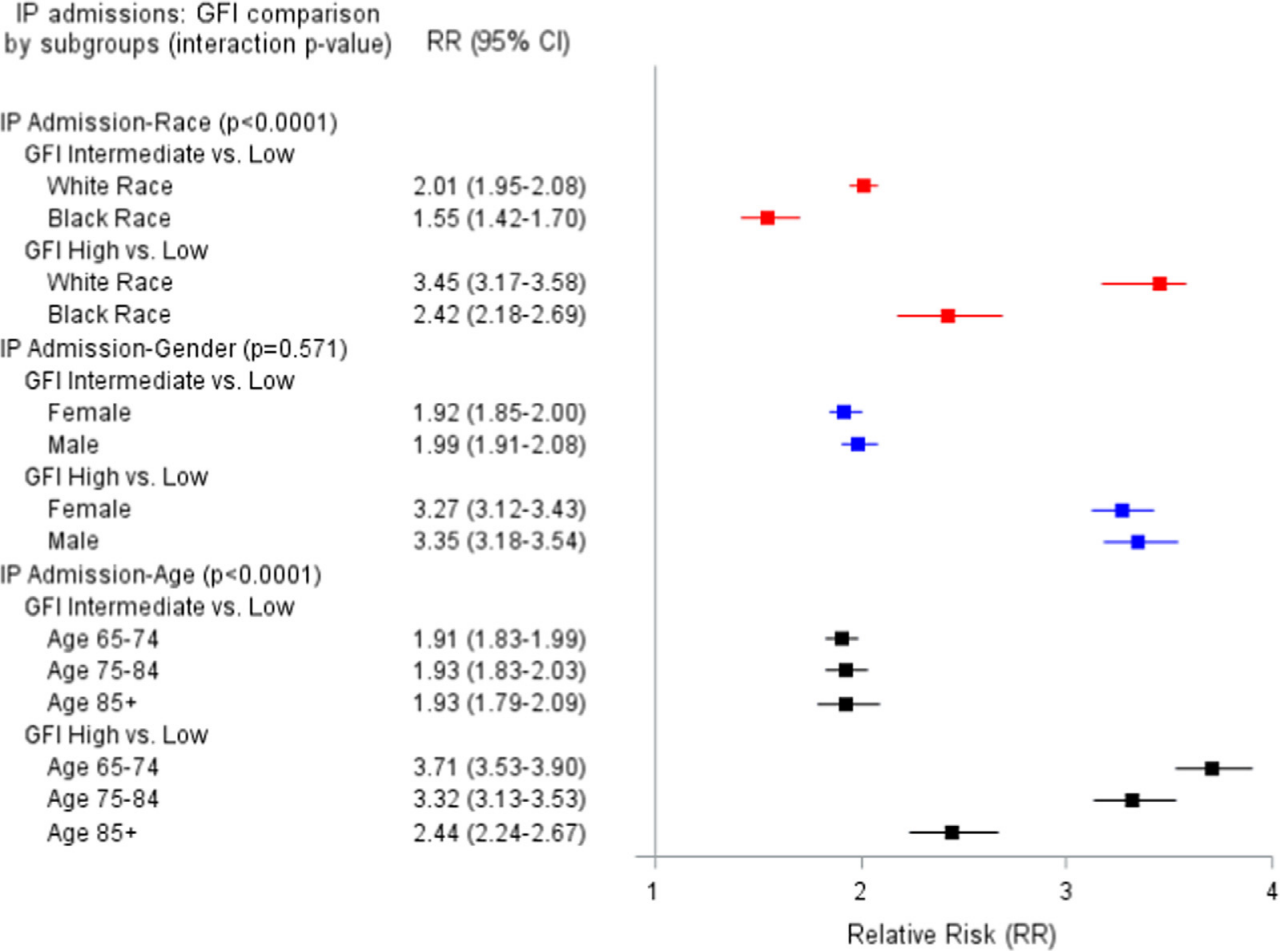
Adjusted Relative risk (95 % CI) of the relationship of Gilbert Frailty Index (GFI) and Inpatient Admissions.

Fig. 1b shows stratified results for ED admissions. Race, gender, and age all modified the association of GFI and ED admission. Among White patients, GFI intermediate and GFI high, compared to GFI low were associated with approximately double and triple the likelihood of ED admissions, respectively (RR=1.97 and RR=2.88, respectively). However, these effects were smaller among Black patients (RR for GFI intermediate=1.58; RR for GFI high = 1.96). Among females, GFI high vs. GFI low was associated with a 2.89 increased risk of ED admissions, while among males, GFI high vs. GFI low was associated with 2.60 times the risk of ED admissions. Finally, for age, GFI high vs. GFI low effects were similar in each age group, but effects were larger as age increased for GFI intermediate vs. GFI low (RR=1.80 for 65–74 years; RR=2.02 for 75–84 years; RR=2.23 for ≥85 years).

**Figure 1b:**
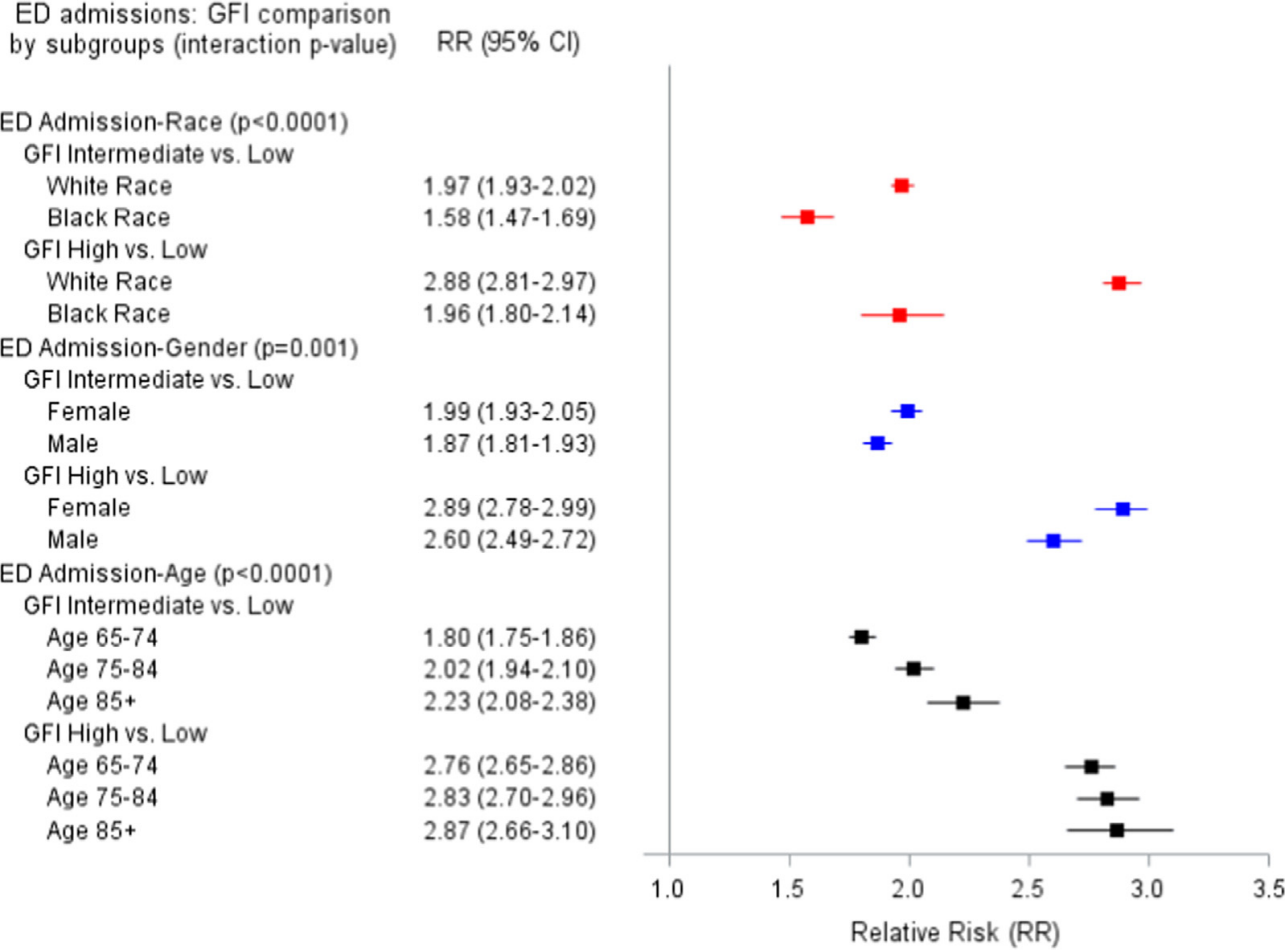
Adjusted Relative risk (95 % CI) of the relationship of Gilbert Frailty Index (GFI) and Emergency Department Admissions.

Fig. 1c shows that only age modified the relationship of GFI and NH admissions. Among the oldest age group (≥85 years), GFI intermediate vs. GFI low was associated with over three times the likelihood of NH admissions (RR=3.16) compared to about twice the risk in patients in the youngest age group (RR=2.28).

**Figure 1c:**
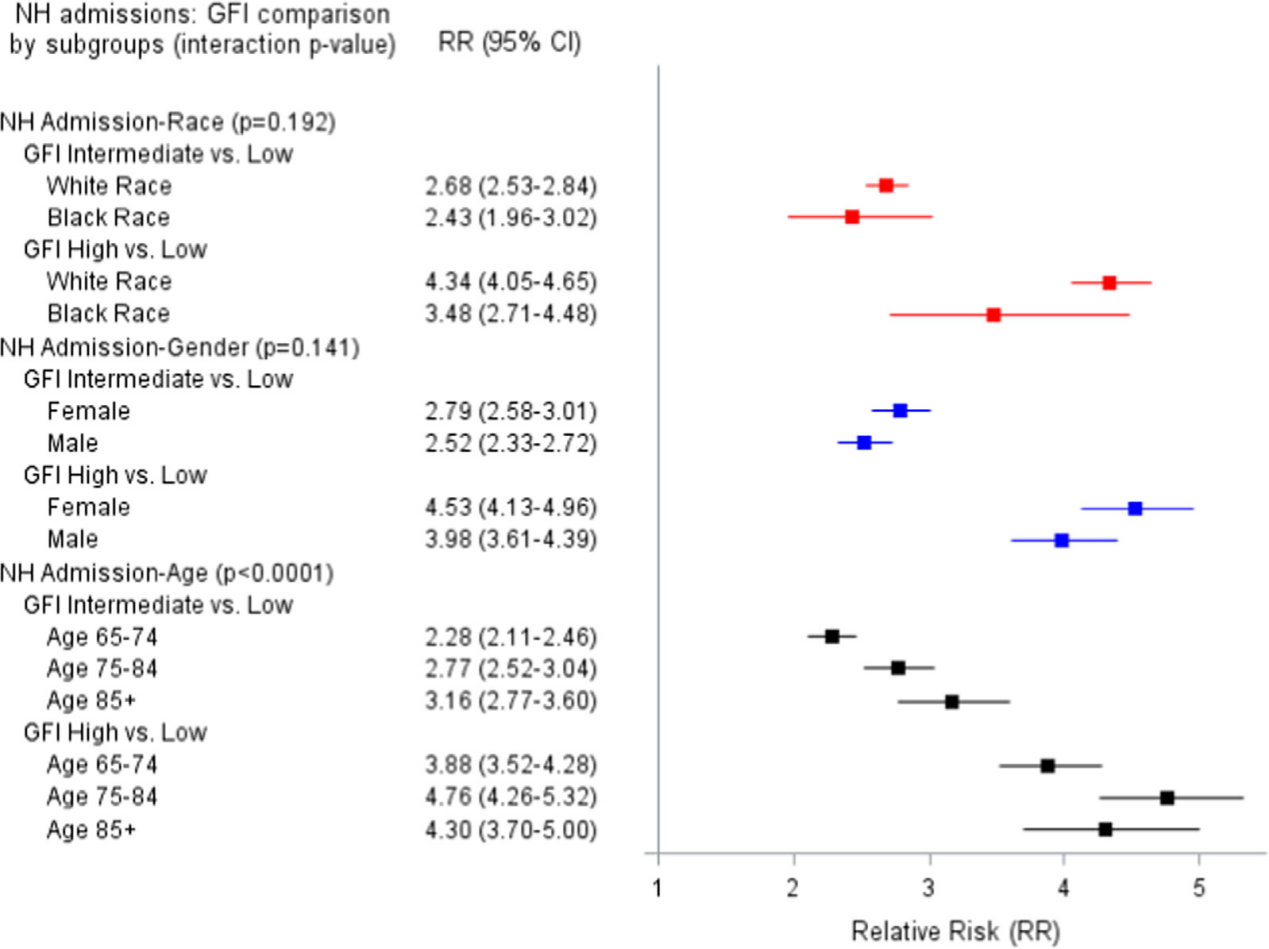
Adjusted Relative risk (95 % CI) of the relationship of Gilbert Frailty Index (GFI) and Nursing Home Admissions.

Finally, Fig. 1d shows that race, gender and age all modified the relationship of GFI and high OCU. White vs. Black patients showed a larger effect for both GFI intermediate (RR=1.64 vs. 1.50) and GFI high (RR=1.63 vs. 1.39) vs. GFI low. Female vs. male patients showed a similar pattern comparing GFI intermediate (RR=1.69 vs. 1.56) and GFI high (RR=1.69 vs. 1.48) vs. GFI low. Finally, patients in the two old age groups (75–84, ≥85) had similar effects of GFI intermediate and high on high OCU; however, patients who were 65–74 had slightly smaller effects.

**Figure 1d:**
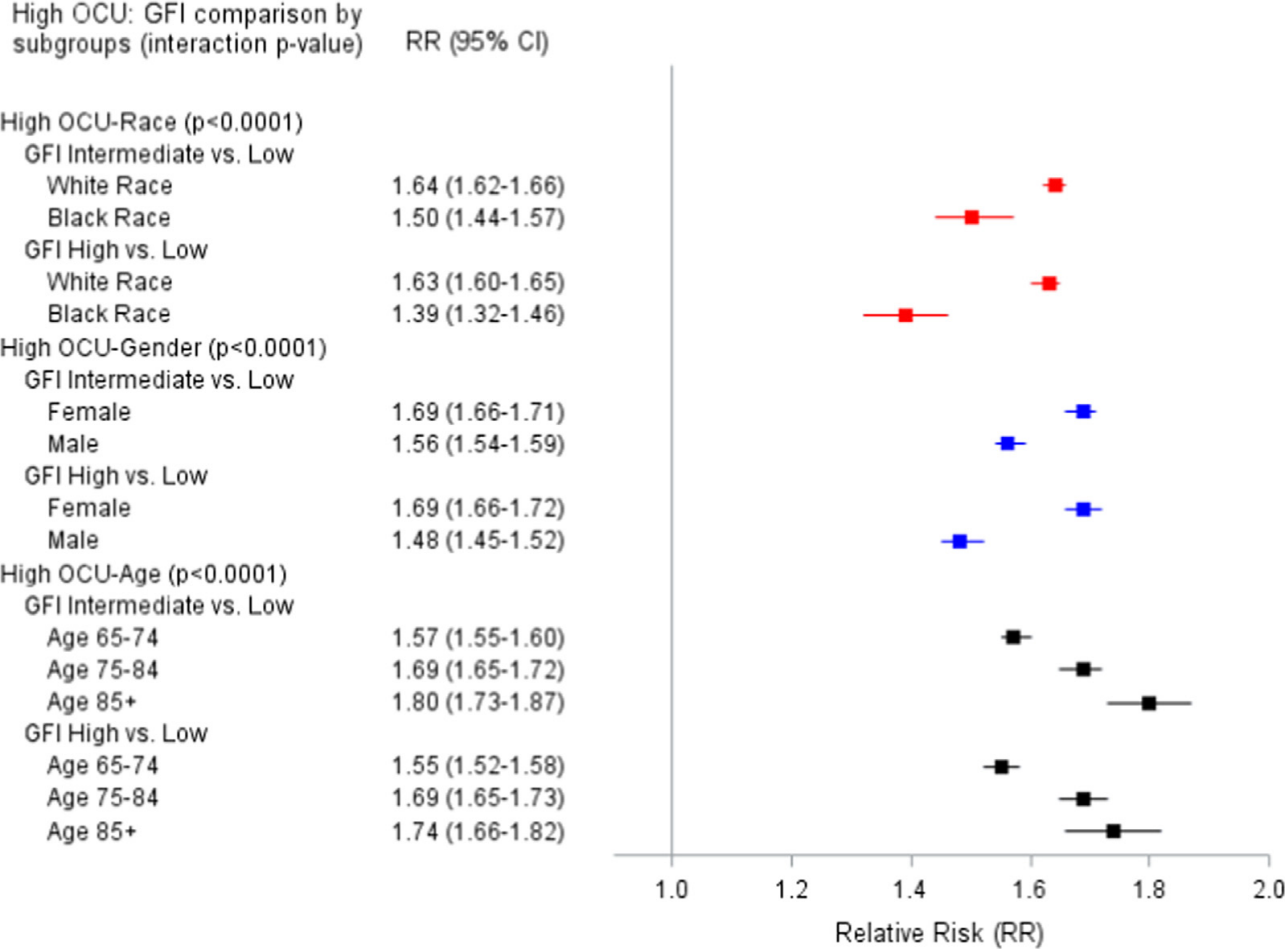
Adjusted Relative risk (95 % CI) of the relationship of Gilbert Frailty Index (GFI) and High Outpatient Clinic Utilization (OCU).

## 4 Discussion

Applying a recently developed frailty index score, the GFI, this study examined the association between hospital frailty index score and healthcare utilization, as well as interactions of these outcomes with demographic characteristics such as age group, race, and gender, among older patients with regular ambulatory office visits. Adjusted models showed patients with a high GFI score had the highest risk for all healthcare utilization outcomes. Significant interactions were observed between age group, race, and gender in the association between GFI risk levels and each outcome. White participants categorized as GFI high consistently showed a higher risk for all healthcare utilization outcomes compared to their Black counterparts with GFI intermediate or low. Female patients with GFI high were more likely to experience ED admissions and high OCU compared to male patients with GFI intermediate or GFI low. Patients aged 85 and older with GFI high or intermediate were at the greatest risk for high OCU, as well as ED and NH admissions, compared to those with GFI low. Interestingly, patients aged 65 to 74 with a high GFI score showed the highest risk of IP admissions compared to older age groups with GFI intermediate or low.

Several aspects of our findings are noteworthy. First, this study extends prior studies on racial variation in frailty. Black older patients had the highest odds of GFI high is consistent with earlier studies using various frailty assessment tools (e.g., phenotype, accumulation of deficits) [31,33,38]. However, we identified a paradox. Adjusted models showed that Black older patients with GFI high had significantly lower odds of healthcare service utilization compared to White patients with the same GFI high. Possible explanations for this discrepancy include differences in socioeconomic status and access to healthcare services, including preventive care. According to the Cumulative Advantage-disadvantage (CAD) theory, social and structural forces contribute to health inequalities, which could explain the cumulative disadvantage related to healthcare access [39,40]. Lifetime perceived discrimination and distrust of healthcare system may also act as a barrier to healthcare access, leading lower rates of screening or preventive care services. These findings highlight the need to improve access to medical care for vulnerable patient populations and reduce disparities in providing better care.

Second, an important takeaway of this finding is that age alone may not drive increased healthcare services. Substantial studies have shown that age is a key factor in the progression of frailty and the corresponding need for healthcare services. However, we found that younger patients (aged 65 to 74) with high GFI showed the highest odds of IP admissions (RR=3.71; CI=3.53–3.90), compared to older patients (RR=3.32; CI=3.13–3.53 for ages 75 to 84, and RR=2.44; CI=2.24–2.67 for ages 85+). Several mechanisms may explain this finding. First, a survivor effect may exist in the oldest patient group aged 85 years, who might have been in better physical conditions and privileged socioeconomic status than those who died earlier [41]. Furthermore, this group of patients may have had better access to preventive healthcare services earlier in their life. Regular access to preventive healthcare services such as routine wellness examinations could help identify the need for acute or urgent care or reduce the risk of adverse outcomes related to chronic conditions [[42], [43], [44]], which enable healthcare providers to detect frailty risk. Ageism or negative age discrimination may be additional explanation as to why older patients were not admitted as frequently. Due to the patient’s age, older patients, especially over 85 years old, may be unfairly assessed and undertreated by medical providers [45]. Clinical guidelines to distinguish among age-related changes, treatable diseases and process of dying should be developed.

Third, the findings underscore the importance of early detection in frailty. The odds of IP admissions decreased with participant age among those with GFI high, while the odds of ED and NH admissions and HCU increased with age. Earlier studies have consistently demonstrated that frailty is associated with healthcare services over time, including hospital admissions and long-term care services [37,46]. However, a recent study highlighted several barriers to routine frailty screening and early detection [47], such as time constraints in clinical practice, a lack of understanding frailty risk, and the absence of consensus or recommendations on frailty screening in clinical settings. Although the GFI was developed for hospitalized patients, its broader applicability is demonstrated to our study population (i.e., older patients with regular ambulatory visits). This indicates that our findings support the potential of GFI as a scalable, EHR-integrated tool for risk stratification among community-dwelling older adults. Improving the recognition of frailty risk by further validation for non-hospitalized patients and developing tailored guidelines for early detection could prevent adverse healthcare outcomes.

Although the results are significant, some limitations should be considered. First, the common limitations of utilizing medical diagnoses still exist despite using diagnostic codes for GFI from a real-world setting. The three categories of GFI were determined by the original developer [23], but these categories may not capture the details from the patient’s perspective. While the GFI was developed in a hospital setting, its application in a broader ambulatory population may limit sensitivity to early functional impairments not captured in diagnostic codes. Future studies should explore complementary assessments or hybrid tools tailored such as functional status, complications with multiple chronic conditions, or continuous ongoing care services from the healthcare system in more detail. Second, compared to other studies on frailty, this study examined the healthcare service outcomes with a five-year period, offering a longer perspective. A longitudinal approach, monitoring various factors related to mortality or health promotion behaviors (e.g., lifestyle factors) [41] could enhance our understanding of frailty trajectories and their relationship with healthcare service outcomes. Lastly, the levels of GFI were identified solely based on the diagnoses from the EHR. If there are undiagnosed or over-diagnosed conditions on the EHR, this could lead to misclassifying the GFI, which, in turn, could bias the findings. Supplemental records, such as algorithms that combine both diagnostic and supporting information, should be developed to overcome this bias [48].

## 5 Conclusion

The strengths of this study include providing evidence on the predictive power of the GFI to healthcare service utilization by identifying older patients at increased risk of hospital use. Integrating the GFI, an EHR-based frailty screening tool, into routine ambulatory care visits may enable healthcare providers to proactively identify high-risk patients and implement early, targeted interventions. Such efforts have the potential to reduce emergency department admissions and nursing home placements. Given the growing diversity and complexity of the geriatric population, early detection of frailty, a common geriatric syndrome, in healthcare settings can be beneficial in reducing adverse outcomes among older patients from diverse backgrounds. Future efforts should prioritize the operationalization of GFI-informed interventions in real-world clinical settings and evaluate their long-term impact on outcomes and costs.

## Acknowledgements

### Author contributions

All authors met criteria for authorship including contributions to the study design, analysis or interpretation of study results, preparation and approval of the final version of the manuscript.

### Sponsor’s role

Funds to maintain the Virtual Data Warehouse came from the Saint Louis University Research Institute. The funder had no role in developing the manuscript or the decision to publish. The Advanced HEAlth Data (AHEAD) Research Institute at Saint Louis University provided data management and biostatistical support.

### Declaration of generative AI and AI-assisted technologies in the writing process

The authors declare that during the writing process of this manuscript, ChatGPT was used to refine sentence structure, suggest alternative phrasing, and correct grammatical errors. All AI-generated content was thoroughly reviewed and edited by the authors to ensure accuracy, originality, and alignment with the intended meaning of the work. The authors take full responsibility for the final content of the manuscript and acknowledge that the AI tool was used as a supporting aid, not as a primary source of authorship.

## CRediT authorship contribution statement

**Jinmyoung Cho:** Writing – review & editing, Writing – original draft, Data curation, Conceptualization. **Joanne Salas:** Writing – review & editing, Writing – original draft, Methodology, Formal analysis, Data curation, Conceptualization. **Jeffery F. Scherrer:** Writing – review & editing, Writing – original draft, Conceptualization. **George Grossberg:** Writing – review & editing, Writing – original draft.

## Declaration of competing interest

The authors declare that they have no known competing financial interests or personal relationships that could have appeared to influence the work reported in this paper.

The author is an Editorial Board Member/Editor-in-Chief/Associate Editor/Guest Editor for *Journal of Adult Development* and was not involved in the editorial review or the decision to publish this article.

## Appendix Supplementary materials

Image, application 1
