# Supplementary material for: A retrospective cohort study on the relationship between frailty and healthcare outcomes
Source: J Frailty Aging. 2025 May 23;14(4):100053. doi: 10.1016/j.tjfa.2025.100053 (PMC12184059; doi:10.1016/j.tjfa.2025.100053)
Supplement: Supplementary file 1 [file mmc1.docx]

**Supplement Figure 1. Sampling**

**Base sample: 3,293,938 with an encounter 1/1/2018 – 12/31/2022**

≥ 1 ambulatory office visit 2018-2021 (n=3,114,234)

≥65 years old at index (1/1/2022) (n=616,703)

≥2 ambulatory office visits 2018-2021, occurring in separate years (n=404,773)

Non-missing demographics

(n=355,266)

- 6,562: Race missing

- 5: Gender missing

Non-institutionalized – no Nursing Home indications 2018-2021 (n=361,829)

**Supplement Table 1. Variable definitions**

| **Variable** | **Definition** |
| --- | --- |
| **Exposure and outcome** | |
| Gilbert Frailty Index (“Frailty score”) | Score ranges from 0-173.2 based on ICD-10-CM diagnostic codes and weights. Measured in the 4-year baseline period (2018-2021)  Low risk < 5; intermediate risk 5-15; high risk > 15  Gilbert T et al. (2018). Development and validation of a hospital frailty risk score focusing on older people in acute care settings using electronic health records: an observational study. *Lancet, 5*(391)(10132): 1775-1782. |
| Inpatient admission | Any encounter type in 2022 listed as acute inpatient admission |
| Emergency department admission | Any encounter type in 2022 listed as emergency department admission |
| Nursing home admission | Any of the following criteria met in 2022:  a) Any encounter type = institutional stay with a subtype of nursing home;  b) CPT codes: 99304-99310, 99315, 99316;  c) ICD-10-CM diagnostic codes: Z02.2, Y92.12 |
| High outpatient clinic utilization | Number of unique ambulatory, outpatient clinic visits (to any specialty). Categorized as high if ≥4 unique encounters in 2022. |
| **Covariates** | |
| Smoking/nicotine dependence | Measured in the 4-year baseline period:  a) ICD-10-CM codes: Z87.891, Z72.0, F17.20x, F17.21x; or  b) current smoker in social history |
| # of drug classes prescribed | Measured in the 4-year baseline period and ranges from 0 to 14.  Count of the number of major categories of classes based on Medi-Span generic product identifier drug group: 01-16 (Anti-infective agents); 17-20 (Biologicals); 21 (Antineoplastic agents); 22-30 (Endocrine and Metabolic); 31-40 (Cardiovascular agents); 41-45 (Respiratory agents); 46-52 (Gastrointestinal agents); 53-56 (Genitourinary products); 57-63 (Central nervous system drugs); 64-71 (Analgesics and Anesthetics); 72-76 (Neuromuscular drugs); 77-81 (Nutritional products); 82-85 (Hematological agents); 86-91 (Topical products) |
| Age at index | Age in years: 65-74, 75-84, ≥85 |
| Race | White, Black, “other” |
| Gender | Male, female |

**Supplement Table 2. Gilbert Frailty Index**

Gilbert T, Neuburger J, Kraindler J, et al. Development and validation of a Hospital Frailty Risk Score focusing on older people in acute care settings using electronic hospital records: an observational study. *Lancet.* 2018;391(10132):1775-1782.

| **ICD-10 code (all codes with associated 1st 3-characters are included)** | **Description** | **points** |
| --- | --- | --- |
| **F00** | Dementia in Alzheimer's disease | 7.1 |
| **G81** | Hemiplegia | 4.4 |
| **G30** | Alzheimer's disease | 4.0 |
| **I69** | Sequelae of cerebrovascular disease (secondary codes) | 3.7 |
| **R29** | Other symptoms and signs involving the nervous and musculoskeletal systems (R29·6 Tendency to fall) | 3.6 |
| **N39** | Other disorders of urinary system (includes urinary tract infection and urinary incontinence) | 3.2 |
| **F05** | Delirium, not induced by alcohol and other psychoactive substances | 3.2 |
| **W19** | Unspecified fall | 3.2 |
| **S00** | Superficial injury of head | 3.2 |
| **R31** | Unspecified haematuria | 3.0 |
| **B96** | Other bacterial agents as the cause of diseases classified to other chapters (secondary code) | 2.9 |
| **R41** | Other symptoms and signs involving cognitive functions and awareness | 2.7 |
| **R26** | Abnormalities of gait and mobility | 2.6 |
| **I67** | Other cerebrovascular diseases | 2.6 |
| **R56** | Convulsions, not elsewhere classified | 2.6 |
| **R40** | Somnolence, stupor and coma | 2.5 |
| **T83** | Complications of genitourinary prosthetic devices, implants and grafts | 2.4 |
| **S06** | Intracranial injury | 2.4 |
| **S42** | Fracture of shoulder and upper arm | 2.3 |
| **E87** | Other disorders of fluid, electrolyte and acid- base balance | 2.3 |
| **M25** | Other joint disorders, not elsewhere classified | 2.3 |
| **E86** | Volume depletion | 2.3 |
| **R54** | Senility | 2.2 |
| **Z50** | Care involving use of rehabilitation procedures | 2.1 |
| **F03** | Unspecified dementia | 2.1 |
| **W18** | Other fall on same level | 2.1 |
| **Z75** | Problems related to medical facilities and other health care | 2.0 |
| **F01** | Vascular dementia | 2.0 |
| **S80** | Superficial injury of lower leg | 2.0 |
| **L03** | Cellulitis | 2.0 |
| **H54** | Blindness and low vision | 1.9 |
| **E53** | Deficiency of other B group vitamins | 1.9 |
| **Z60** | Problems related to social environment | 1.8 |
| **G20** | Parkinson's disease | 1.8 |
| **R55** | Syncope and collapse | 1.8 |
| **S22** | Fracture of rib(s), sternum and thoracic spine | 1.8 |
| **K59** | Other functional intestinal disorders | 1.8 |
| **N17** | Acute renal failure | 1.8 |
| **L89** | Decubitus ulcer | 1.7 |
| **Z22** | Carrier of infectious disease | 1.7 |
| **B95** | Streptococcus and staphylococcus as the cause of diseases classified to other chapters | 1.7 |
| **L97** | Ulcer of lower limb, not elsewhere classified | 1.6 |
| **R44** | Other symptoms and signs involving general sensations and perceptions | 1.6 |
| **K26** | Duodenal ulcer | 1.6 |
| **I95** | Hypotension | 1.6 |
| **N19** | Unspecified renal failure | 1.6 |
| **A41** | Other septicaemia | 1.6 |
| **Z87** | Personal history of other diseases and conditions | 1.5 |
| **J96** | Respiratory failure, not elsewhere classified | 1.5 |
| **X59** | Exposure to unspecified factor | 1.5 |
| **M19** | Other arthrosis | 1.5 |
| **G40** | Epilepsy | 1.5 |
| **M81** | Osteoporosis without pathological fracture | 1.4 |
| **S72** | Fracture of femur | 1.4 |
| **S32** | Fracture of lumbar spine and pelvis | 1.4 |
| **E16** | Other disorders of pancreatic internal secretion | 1.4 |
| **R94** | Abnormal results of function studies | 1.4 |
| **N18** | Chronic renal failure | 1.4 |
| **R33** | Retention of urine | 1.3 |
| **R69** | Unknown and unspecified causes of morbidity | 1.3 |
| **N28** | Other disorders of kidney and ureter, not elsewhere classified | 1.3 |
| **R32** | Unspecified urinary incontinence | 1.2 |
| **G31** | Other degenerative diseases of nervous system, not elsewhere classified | 1.2 |
| **Y95** | Nosocomial condition | 1.2 |
| **S09** | Other and unspecified injuries of head | 1.2 |
| **R45** | Symptoms and signs involving emotional state | 1.2 |
| **G45** | Transient cerebral ischaemic attacks and related syndromes | 1.2 |
| **Z74** | Problems related to care-provider dependency | 1.1 |
| **M79** | Other soft tissue disorders, not elsewhere classified | 1.1 |
| **W06** | Fall involving bed | 1.1 |
| **S01** | Open wound of head | 1.1 |
| **A04** | Other bacterial intestinal infections | 1.1 |
| **A09** | Diarrhoea and gastroenteritis of presumed infectious origin | 1.1 |
| **J18** | Pneumonia, organism unspecified | 1.1 |
| **J69** | Pneumonitis due to solids and liquids | 1.0 |
| **R47** | Speech disturbances, not elsewhere classified | 1.0 |
| **E55** | Vitamin D deficiency | 1.0 |
| **Z93** | Artificial opening status | 1.0 |
| **R02** | Gangrene, not elsewhere classified | 1.0 |
| **R63** | Symptoms and signs concerning food and fluid intake | 0.9 |
| **H91** | Other hearing loss | 0.9 |
| **W10** | Fall on and from stairs and steps | 0.9 |
| **W01** | Fall on same level from slipping, tripping and stumbling | 0.9 |
| **E05** | Thyrotoxicosis [hyperthyroidism] | 0.9 |
| **M41** | Scoliosis | 0.9 |
| **R13** | Dysphagia | 0.8 |
| **Z99** | Dependence on enabling machines and devices | 0.8 |
| **U80** | Agent resistant to penicillin and related antibiotics | 0.8 |
| **M80** | Osteoporosis with pathological fracture | 0.8 |
| **K92** | Other diseases of digestive system | 0.8 |
| **I63** | Cerebral Infarction | 0.8 |
| **N20** | Calculus of kidney and ureter | 0.7 |
| **F10** | Mental and behavioural disorders due to use of alcohol | 0.7 |
| **Y84** | Other medical procedures as the cause of abnormal reaction of the patient | 0.7 |
| **R00** | Abnormalities of heart beat | 0.7 |
| **J22** | Unspecified acute lower respiratory infection | 0.7 |
| **Z73** | Problems related to life-management difficulty | 0.6 |
| **R79** | Other abnormal findings of blood chemistry | 0.6 |
| **Z91** | Personal history of risk-factors, not elsewhere classified | 0.5 |
| **S51** | Open wound of forearm | 0.5 |
| **F32** | Depressive episode | 0.5 |
| **M48** | Spinal stenosis (secondary code only) | 0.5 |
| **E83** | Disorders of mineral metabolism | 0.4 |
| **M15** | Polyarthrosis | 0.4 |
| **D64** | Other anaemias | 0.4 |
| **L08** | Other local infections of skin and subcutaneous tissue | 0.4 |
| **R11** | Nausea and vomiting | 0.3 |
| **K52** | Other noninfective gastroenteritis and colitis | 0.3 |
| **R50** | Fever of unknown origin | 0.1 |
